# Clustering of *Streptococcus thermophilus* Strains to Establish a Relation between Exopolysaccharide Characteristics and Gel Properties of Acidified Milk

**DOI:** 10.3390/foods8050146

**Published:** 2019-04-30

**Authors:** Georg Surber, Susann Mende, Doris Jaros, Harald Rohm

**Affiliations:** Institute of Natural Materials Technology, Technische Universität Dresden, 01062 Dresden, Germany; susann.mende@tu-dresden.de (S.M.); doris.jaros@tu-dresden.de (D.J.); harald.rohm@tu-dresden.de (H.R.)

**Keywords:** yoghurt, exopolysaccharides, fermented dairy products, gel formation, cluster analysis

## Abstract

In situ produced extracellular polysaccharides (EPS) from lactic acid bacteria are generally known to affect the texture of fermented dairy products; however, the interplay between EPS and product properties is still poorly understood. The aim of this study was to establish a relationship between concentration and properties of EPS, and gel formation of milk analysed by noninvasive Multispeckle Diffusing Wave Spectroscopy. Twenty *Streptococcus thermophilus* strains were classified with respect to EPS concentration (8–126 mg GE/kg) and ropiness (thread length: 15–80 mm). Five groups identified by cluster analysis demonstrate the high strain-to-strain variability even within one species of lactic acid bacteria. Results from acidification and gelation experiments averaged per cluster indicate that fermentation time and gel stiffness is higher for strains that produce ropy EPS. A further increase in gel stiffness was detected for strains that also produced cell-bound EPS, which underlines the importance of both ropy and cell-bound EPS for improving acid gel properties. The results may be helpful for a proper selection of EPS-producing starter cultures.

## 1. Introduction

In situ produced extracellular polysaccharides (EPS) from lactic acid bacteria (LAB) are generally known to have an impact on the texture and, consequently, on sensory properties of fermented foods. Bacteria strains with the ability of synthesising EPS are utilised in the manufacture of a broad range of products, especially in the cereal [1] and dairy sector [2]. For the latter, positive effects were observed for viscosity, thickness and creaminess of yoghurt [3,4], and EPS also reduce the protein particle growth during fresh cheese manufacture [5]. However, the interplay between EPS properties and product microstructure is still poorly understood [2].

Recent reviews [2,6] highlight the diversity of EPS with respect to their chemical structure (e.g., homo- or heteropolysaccharides, linear or branched molecules) and their macromolecular properties (e.g., molecular mass, stiffness, charge). EPS from LAB are often distinguished by the degree of ropiness they induce in the product, and by their location: whereas free EPS (fEPS) are released into the surrounding medium, cell-bound EPS (cEPS) remain attached to the bacteria cells.

A common method to assess ropiness is to visually evaluate the slimy, thread-forming character of the fermented medium [7,8]. Ropy EPS were also identified by measuring viscosity of the cell-free sera [9], the efflux time of broth cultures from microhaematocrit capillaries [8], or extensibility of acid gel suspensions [10,11]. Different microscopy techniques such as counter- or negative-staining [12,13], autofluorescence [14] or transmission electron microscopy [15] were applied to visualise cEPS, and staining with fluorescence dyes or their conjugates to visualise both fEPS and cEPS [16,17]. To discriminate between EPS-producing strains, several workers determined monomer composition [13,18,19], chemical structure of EPS [20,21,22], EPS concentration [9,13] or macromolecular properties of aqueous EPS solutions [23,24]. Rheological methods and sensory evaluations were extensively used to investigate the impact of EPS producers on the texture of dairy products to facilitate the selection of appropriate strains [4,23,25].

Although extensive research has been carried out on the general impact of EPS on the texture of fermented dairy products, only a few studies related properties of the respective EPS to the resulting product characteristics. Hassan et al. [26] focused on EPS type and determined higher apparent viscosity in products with ropy fEPS than in products containing both nonropy fEPS and cEPS. Mende et al. [23] reported that ropiness of EPS from *Streptococcus thermophilus* is mainly caused by intrinsic properties (e.g., composition, branching), and that ropiness is linked to increased apparent viscosity of stirred acidified milk. For EPS with identical structure from two *S. thermophilus* strains, Faber et al. [27] determined higher medium viscosity for EPS with higher molecular mass. A positive correlation between EPS molecular mass and fermented milk viscosity was also found for 28 *Lactobacillus* strains [28]. Vaningelgem et al. [24] investigated 26 strains of *S. thermophilus* and proposed a classification by molecular mass and monomer composition for further studies of function–texture relationship. Monomer composition was also used by Mozzi et al. [13] to distinguish between EPS producers of different species. Recently, there has been an increasing interest in identifying the relationship between gene clusters and textural properties [10,29,30]. 

The impact of different EPS starts during gelation and might already alter the gel formation behaviour of milk [2]. Hassan et al. [31] investigated acid gelation of milk with three yoghurt cultures which produced different types of EPS, and detected similar gelation profiles but an earlier gelation onset for strains that produced nonropy fEPS and cEPS compared to ropy fEPS. Purwandari et al. [32] observed higher stiffness of gels with nonropy fEPS and cEPS compared to gels with ropy fEPS and cEPS, whereas Bullard et al. [33] determined lower gel stiffness for gels with nonropy fEPS and cEPS. Mende et al. [23] detected higher stiffness for gels made with *S. thermophilus* strains that produced low amounts of EPS compared to strains that produced high amounts. Because of the great diversity of strains and the corresponding EPS, there is still uncertainty concerning the effects of EPS on gel formation. 

The aim of our study was to establish a relationship between EPS properties and gelation characteristics of acidified milk by clustering 20 *S. thermophilus* strains on the basis of ropiness and EPS concentration. The results may support the prediction of product characteristics influenced by certain EPS properties.

## 2. Materials and Methods

### 2.1. Preparation of Strains

Twenty single strains of *S. thermophilus* (ST): ST-1C, D, E, G, H, I, K; ST-2A, B, C, D, E, F, G, H, I, K, L, M; and the EPS-negative DSMZ20259 [34] (Deutsche Sammlung für Mikroorganismen und Zellkulturen GmbH, Braunschweig, Germany) were stored as cryocultures at −80 °C. Glass pellets were used to prepare precultures by anaerobic incubation (48 h, 40 °C) in a semidefined medium with 200 mM lactose [23].

### 2.2. Production of Acid Gel Suspensions

Stirred acid gels were prepared in laboratory scale based on the process described previously [35]. Reconstituted skim milk (RSM) was prepared by dissolving low-heat skim powder in demineralised water to achieve a dry matter of 10% (*w/w*). RSM was heated to 90 °C for 10 min in a water bath, subsequently cooled to 40 °C and 0.45 L was transferred into plastic vessels. Aerobic cultivation was carried out after inoculation with 1% (*v/v*) preculture in a water bath at 40 °C for 24 h. During cultivation, acidification was continuously monitored using a 6-channel pH logger (EA Instruments Ltd., Wembley, UK). Time until pH 4.6 (t_pH = 4.6_) and pH at the end of fermentation (pH_t = 24 h_) were evaluated. Subsequently, the milk gels were broken using a perforated plunger (d = 78 mm) [36] which was moved upwards and downwards for 2 × 30 s, and then stirred at 400 rpm for 2 min with a 3-wing propeller blade (d = 70 mm) mounted on a Eurostar power control-visc stirrer (IKA GmbH & Co. KG, Staufen, Germany). 

The fermentation medium used for ropiness determination was RSM supplemented with 1% (*w/v*) tryptically digested casein peptone and 0.5% (*w/v*) yeast nitrogen base (RSM_suppl_) that was autoclaved at 110 °C for 10 min [23]. After cultivation, the gels were broken using the perforated plunger only.

All acid gels suspensions from RSM and RSM_suppl_ were produced in duplicate. Analysis of EPS concentration was started directly after fermentation. All other measurements were performed after three days storage at 6 °C. All results are expressed as arithmetic means from duplicate measurements.

### 2.3. Analysis of Acid Gel Suspensions

EPS isolation and quantification was performed as described by Mende et al. [23]. In brief, acid gel suspensions adjusted to pH 7.5 with 1 M NaOH were incubated with 0.25 mL of a 0.5% (*w/v*) Pronase E solution (Sigma-Aldrich Chemie GmbH, Steinheim, Germany) at 37 °C for approximately 18 h to cleave the proteins. After adjusting to 9% (*w/v*) trichloroacetic acid (TCA) and thermal treatment (10 min at 90 °C), cells and proteins were removed by centrifugation (19,000× *g*, 15 min, 6 °C). The pellets were resuspended in 0.5 mL 10% (*w/v*) TCA and centrifuged again. Two volumes of chilled acetone (6 °C) were added to the pooled supernatants. On the next day, the EPS precipitated by the solvent were collected through centrifugation (19,000× *g*, 15 min, 6 °C), dissolved in water, dialysed (molecular mass cut-off 8–10 kDa; Carl Roth GmbH & Co. KG, Karlsruhe, Germany) for 48 h against demineralised water, and freeze-dried (Alpha 1-2, Martin Christ Gefriertrocknungsanlagen GmbH, Osterode, Germany). EPS concentration, determined by the phenol-sulphuric acid method [37] in duplicate, is expressed as milligram glucose equivalent (GE) per kilogram medium. Fresh media served as blank and their absorbance was subtracted from those of the fermented samples.

Cell-free milk serum was obtained by centrifugation of the acid gel suspension at 19,000× *g* and 6 °C for 15 min. Dynamic viscosity of the serum was determined with a LOVIS rolling ball microviscometer (Anton Paar GmbH, Graz, Austria). The sample was filled into a glass capillary of 1.59 mm diameter and conditioned to 20 °C. Measurements were performed at a capillary angle of 70° with two independently prepared sera in six-fold. Dynamic viscosity was calculated from the rolling time of a gold-coated stainless steel ball (d = 1.5 mm, ρ = 7.88 g/mL) through the capillary [38] and the density of the serum, which was determined by the oscillating U-tube method. Mean density of 1.029 ± 0.001 g/mL and 1.032 ± 0.001 g/mL were used for RSM and RSM_suppl_, respectively.

Negative staining with Indian ink was used to visualize EPS [13]. Ink particles are not able to penetrate into the polymeric material, so that EPS capsules appear as clear zones around the cells. Cell-bound EPS producers are indicated by ‘+’ after strain name.

Ropiness of the acid gel suspensions with RSM_suppl_ was measured with a TA-XT.plus analyser (Stable Microsystems Ltd., Godalming, UK). Thirty ±1 g gel suspension was transferred into a petri dish, and a cylindrical probe (d = 40 mm) mounted to the force transducer was lowered until full contact with the sample surface was achieved. The crosshead was then moved upwards at a velocity of 40 mm/s, and each experiment was recorded at 30 frames per second using a digital video camera. Time sequences of the video were analysed to determine the time span until the thread teared. This time measure and crosshead velocity were used to calculate the maximum length of the thread (L_thread_).

### 2.4. Analysis of Gelation Behaviour

Gel formation was analysed by Multispeckle Diffusing Wave Spectroscopy (MS-DWS) using a Rheolaser^TM^ MASTER (Formulaction SA, Toulouse, France). Twenty mL inoculated RSM was transferred into glass vials that were placed in the measurement cell. Time-based backscattering of a λ = 650 nm laser was measured using a multi-pixel detector, and internally converted into curves of Mean Square Displacements (MSD) over decorrelation time by instrument software V1.4 (Formulaction SA, Toulouse, France). Temperature in the measuring cell was kept constant at 40 °C using a Peltier element. The elasticity index (EI) was determined from the inverse mean MSD at short decorrelation times (<0.1 s). The EI at pH 4.6 (EI_pH = 4.6_), gelation onset (t_Onset_) determined at EI = 10^−3^ nm^2^ and the corresponding pH at gelation onset (pH_Onset_) taken from the acidification profiles are further used as measures. 

### 2.5. Statistical Data Evaluation

SAS^®^ University Edition 6p.2 (SAS^®^ Institute, Cary, NC, USA) was used for Euclidian distance cluster analysis using Ward linkage on averaged L_thread_ and EPS concentration, and for univariate ANOVA. Unless stated otherwise, data are expressed as arithmetic mean ± half deviation range (*n* = 2) or arithmetic mean ± standard deviation (*n* > 2).

## 3. Results and Discussion

### 3.1. Exopolysaccharides in Acid Gel Suspensions

The concentration of EPS in acid gel suspensions was highly strain-dependent and varied from 8–126 mg GE/kg (Table 1). A similar range of 10–235 mg/L was found when milk was fermented with various starter cultures [4,9,23,39].

Dynamic viscosity of the separated serum ranged from 1.30–1.51 mPa·s. A higher serum viscosity, observed for e.g., ST-2K.+, must be attributed to remaining fEPS as the cEPS are separated with the cells. Serum viscosity was found to be related to yoghurt viscosity [40,41,42]. In agreement with observations of Ren et al. [9] on LAB wild strains isolated from fermented vegetables, serum viscosity was not correlated with EPS concentration (*r* = 0.05, *p* > 0.05). These findings support the statement that texture and product viscosity can not only be explained by EPS concentration when different EPS producers are considered [25,43,44]. However, for EPS of a specific strain, a higher concentration was linked to higher product viscosity [45].

**Figure 1:**
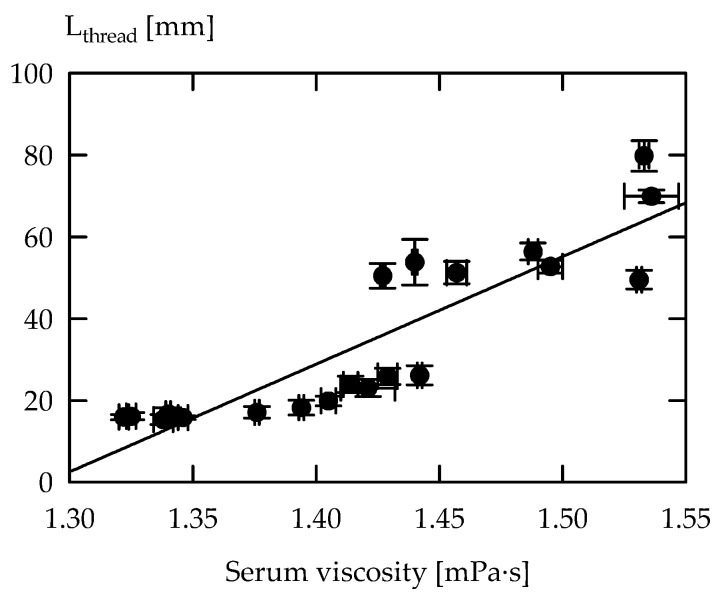
Correlation plot of length of thread (L_thread_) vs. serum viscosity of acid gel suspensions from supplemented reconstituted skim milk fermented with *S. thermophilus* strains (*r* = 0.88, *p* < 0.05).

To quantify ropiness, Mozzi et al. [46] used a pipette, and denoted EPS as ropy when strings longer than 6 mm were observed. Other researchers who classified EPS ropiness on the basis of thread formation and extensibility used a so-called siphon test [4], an extensional rheometer [10], or a universal testing machine [11]. In the latter approach, Hess et al. [11] expanded the samples between two parallel plates at a velocity of 10 mm/s, and observed L_thread_ of 17–53 mm for milk fermented with different starter cultures. A higher thread length is an indicator for a more extensible product. In the current study, we decided to apply a higher velocity of 40 mm/s, and RSM_suppl_ was chosen as fermentation medium for this test. This resulted in an extended range of L_thread_ (e.g., ST-1E: 26 mm for RSM and 10 mm/s; RSM_suppl_: 31 mm for 10 mm/s and 80 mm for 40 mm/s), and L_thread_ correlated significantly with the serum viscosity of acid gel suspensions made from RSM_suppl_ (Figure 1). The findings suggest that serum viscosity can be used as an indicator for ropiness. It cannot be completely excluded that the supplementation of RSM may affect EPS formation, but viscosity of the sera obtained after fermentation in RSM and RSM_suppl_ correlated significantly (*r* = 0.84, *p* < 0.05).

### 3.2. Strain Classification

**Figure 2:**
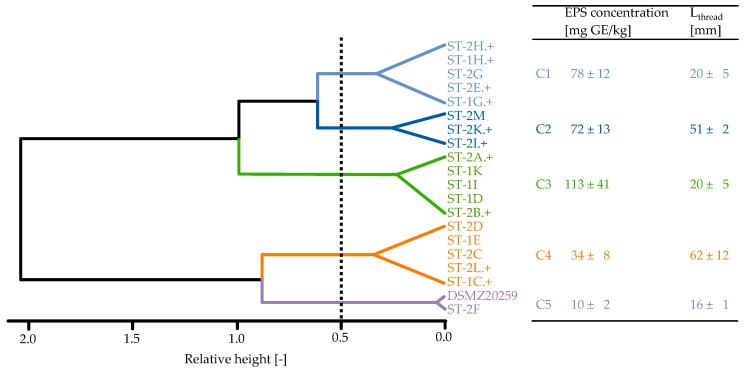
Cluster plot of Euclidian distance cluster analysis using Ward linkage on length of thread (L_thread_) and EPS concentration of acid gel suspensions. Inserted table represents average data of L_thread_ and EPS concentration for each cluster (C). A ‘+’ after the strain code indicates microscopic evidence of cell-bound EPS.

In cluster analysis performed on EPS concentration and L_thread_, five distinct clusters (C) were identified (Figure 2). As serum viscosity correlated with L_thread_ (see Figure 1), it was excluded from cluster analysis. At the first cluster branch there is a clear separation into strains showing high (C1–C3) or low EPS concentration (C4–C5). Strains producing low amounts of EPS are well separated by L_thread_ into clusters C4 and C5. The strains ST-2F and DSMZ20259 of C5 (low EPS concentration, low L_thread_) can be denoted as EPS negative. Cluster 4 represents strains producing low amounts of EPS, but evoking high ropiness in acid gel suspensions. Strains in C1–C3 which produced high amounts of EPS can be further distinguished by EPS concentration at the second branch (approx. 113 vs. 75 mg GE/kg); the third branch allows distinguishing between C1 and C2 by L_thread_. L_thread_ of the strains in clusters C1 and C3 was similar to that of the EPS-negative strains (C5), indicating nonropy fEPS. The high strain-to-strain variability even within one species of LAB is clearly evident from this classification approach. As highly ropy strains were found in both C2 and C4, it is obvious that EPS concentration is not the only factor that contributes to ropiness. Strains producing cEPS were present in all clusters, except for the EPS-negative strain (C5).

### 3.3. Effect of Clusters on Gelation Behaviour

In recent studies, Multispeckle Diffusing Wave Spectroscopy was used to investigate acid-induced [47] and rennet-induced gelation of skim milk [48]. The elasticity index recorded by the instrument correlates with the storage modulus from small deformation rheology and can therefore be taken as an indicator for gel stiffness [47,49].

**Figure 3:**
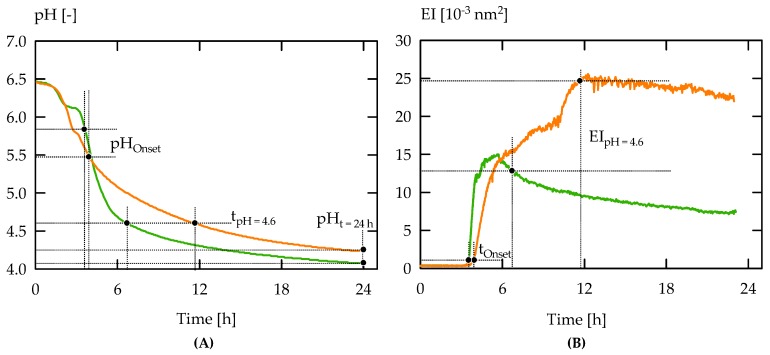
pH (**A**) and elasticity index (EI) (**B**) during acidification of reconstituted skim milk with ST-2B.+ (green) and ST-2C (orange). Parameters obtained are time until pH 4.6 (t_pH = 4.6_), pH at end of fermentation (pH_t = 24 h_), gelation onset at EI = 10^−3^ nm^2^ (t_Onset_) and corresponding pH (pH_Onset_), EI at pH 4.6 (EI_pH = 4.6_). Curves are representative runs (see Figure 4 for statistical analysis).

Figure 3 compares the acidification and gelation curves for ST-2C, producing ropy fEPS, from cluster 4, and for ST-2B.+, producing nonropy fEPS and cEPS, assigned to C3. Apart from t_pH = 4.6_ and pH_t = 24 h_, gelation onset time at EI = 10^−3^ nm^2^ (t_Onset_), the corresponding pH (pH_Onset_), and EI at pH 4.6 (EI_pH = 4.6_) were read from the MS-DWS curves. The acidification activity was less pronounced for ST-2C (both t_pH = 4.6_ and pH_t = 24 h_ were higher), which also affected gelation behaviour (t_Onset_ and EI_pH = 4.6_ were higher, and pH_Onset_ was lower as compared to ST-2B.+).

**Figure 4:**
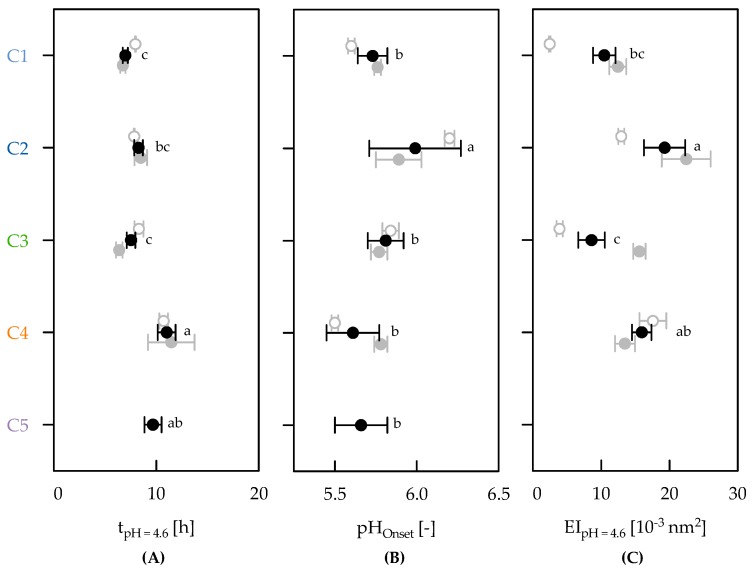
Cluster-specific average time until pH 4.6 (t_pH = 4.6_) (**A**), pH at gelation onset (pH_Onset_) (**B**) and elasticity index at pH 4.6 (EI_pH = 4.6_) (black, **C**). Mean values in the same plot with different superscripts differ significantly at *p* < 0.10. To study the influence of cell-bound EPS (cEPS), data is also displayed for non-cEPS (grey, open) and cEPS producers (grey, closed). EI_pH = 4.6_ of C5 is not included due to gel shrinkage. Bars indicate standard error of the mean.

In the next step, the mentioned parameters were averaged per cluster. As a significant correlation was found between t_pH = 4.6_ and pH_t = 24 h_ and between t_pH = 4.6_ and t_Onset_ (*r* = 0.82 and *r* = 0.84, respectively; *p* < 0.10), pH_t = 24 h_ and t_Onset_ were not considered in further analysis. The remaining data for the respective clusters are shown in Figure 4. Time to reach pH 4.6 was higher for strains producing ropy fEPS (C2 and C4) and for the EPS negative strains (C5) than for nonropy fEPS producing strains (C1 and C3), indicating slower acidification. A higher t_pH = 4.6_ was therefore necessary for strains that produced low amounts of EPS or none (C4, C5), see also Mende et al. [23]. pH at gelation onset was significantly higher for strains producing higher amounts of EPS and ropy fEPS (C2) than for the low EPS (C4, C5) and nonropy fEPS strains (C1, C3). During gel formation, higher amounts of polysaccharides may affect attraction forces between larger casein micelles because of larger phase separating regions [50]. For the strains of C1–C4 which produced also cEPS, pH_Onset_ was similar to that of non-cEPS producers (C5), while Hassan et al. [31] detected an earlier gelation onset for cEPS producers. However, the observed pH_Onset_ in this study was generally higher than earlier findings based on oscillatory rheology [23,51], which was also found by Kristo et al. [52]. Generally, the gelation pH of heated milks is close to the isoelectric point of whey proteins (pH ~5.3) [53]. An earlier gelation onset observed by MS-DWS was recently explained by the noninvasive particle tracking observations in the measurements [49].

A higher gel stiffness as indicated by EI_pH = 4.6_ was observed for the ropy fEPS producers in C2 and C4, and can be explained by reduced particle motion at the measurement point in the MS-DWS cuvettes (height = 24 mm) [47]. Data of the EPS negative strains are missing because of inhomogeneities due to excessive gel shrinkage [47] because of syneresis, which was found to be higher for acid gels with EPS-negative strains [23,33]. The impact of ropy EPS on gel stiffness was frequently addressed after performing oscillatory rheology [23,31,32,33,39,45]. For the strains producing high amounts of EPS (C1–C3), the results further indicate that the presence of cEPS is responsible for the higher gel stiffness, which might be explained by a high water-binding ability similar to ropy fEPS [38]. For gels containing ropy fEPS, the higher stiffness might be attributed to interactions between EPS and protein or the prolonged fermentation (higher t_pH = 4.6_), which affects microstructure formation and the incorporation of EPS in the protein network [54,55]. To result in similar acidification rate, Khanal and Lucey [51] supplemented milk with different amounts of peptone, and found differences in gel stiffness between strains that produced EPS with different chemical structure and molecular mass. However, the supplementation of any substrate with a protein fraction is known to enhance EPS production [56], and resulted in a higher storage modulus of whey permeate fermented with EPS-producing strains [57]. Hassan et al. [31] used three yoghurt cultures showing a similar acidification time and explained differences in gel stiffness by the type of EPS. They detected a higher stiffness of acid gels that contained nonropy fEPS and cEPS, compared to gels with ropy fEPS and cEPS. This is in contrast to Bullard et al. [33] who were not able to distinguish effects on product properties that were induced by different types of EPS. For single strains, a higher stiffness for nonropy fEPS and cEPS was observed by Purwandari et al. [32], while Gentes et al. [58] detected a higher stiffness in gels with ropy fEPS. Our findings point on the importance of both ropy fEPS and cEPS for the improvement of acid gel properties.

Finally, we observed a moderate correlation between EI_pH = 4.6_ and the EPS concentration for the different clusters (*r* = 0.82, *p* < 0.10). This correlation was also reported for *S. thermophilus* strains when adding isolated fractions of EPS at increasing concentration to milk prior to acidification [45], or for in situ produced EPS in yoghurt gels [4,32]. Mende et al. [2] summarised that the relation between EPS concentration and product properties is still a controversial issue, while other factors such as macromolecular and structural properties of EPS or interactions between EPS and protein have to be considered.

## 4. Conclusions

Exopolysaccharides from *S. thermophilus* strains showed a major effect on the physical properties of acid gel suspensions. The length of thread as the indicator of ropiness was found to correlate well with the viscosity of the cell-free serum, but was not clearly related to EPS concentration. We therefore highlight the benefits of detecting serum viscosity as an indicator for the degree of ropiness.

The high strain-to-strain variability even within one species of LAB is clearly evident from cluster analysis performed on EPS concentration and ropiness. Highly ropy strains were found to produce either high or low amounts of EPS. Strains also producing cEPS were represented in all clusters, except EPS-negative strains. Results from acidification and gelation curves averaged per cluster indicated increased fermentation time and gel stiffness for strains producing ropy fEPS than for nonropy fEPS producers. A further increase in stiffness was detected for strains also producing cEPS, which point to the importance of both ropy fEPS and cEPS for the improvement of the texture of fermented dairy products. A moderate correlation was found between gel stiffness and EPS concentration; however, other factors have to be considered. It is suggested to determine texture (e.g., syneresis, sensory) and physicochemical properties (e.g., molecular mass) in combination with biochemical and structural characterisation for the examination of correlations between these characteristics and their impact on a product.

## Figures and Tables

**Table 1:** Exopolysaccharide (EPS) concentration in reconstituted skim milk fermented with *Streptococcus thermophilus* (ST) strains, and corresponding serum viscosity.

Strain ^1^	EPS Concentration (mg GE/kg) ^2^	Serum Viscosity (mPa·s)
ST-1C.+	25 ± 4.9 ^fg^	1.34 ± 0.004 ^f^
ST-1D	106 ± 36.3 ^b^	1.30 ± 0.006 ^h^
ST-1E	25 ± 1.1 ^fg^	1.42 ± 0.020 ^d^
ST-1G.+	80 ± 18.2 ^c^	1.40 ± 0.005 ^e^
ST-1H.+	59 ± 6.6 ^d^	1.32 ± 0.004 ^g^
ST-1I	110 ± 7.7 ^b^	1.35 ± 0.001 ^f^
ST-1K	125 ± 16.7 ^a^	1.31 ± 0.008 ^gh^
ST-2A.+	118 ± 17.4 ^ab^	1.43 ± 0.008 ^cd^
ST-2B.+	106 ± 9.9 ^b^	1.45 ± 0.006 ^c^
ST-2C	33 ± 2.3 ^ef^	1.42 ± 0.005 ^d^
ST-2D	44 ± 8.8 ^e^	1.49 ± 0.006 ^b^
ST-2E.+	87 ± 13.0 ^c^	1.44 ± 0.002 ^cd^
ST-2F	8 ± 2.4 ^h^	1.30 ± 0.001 ^h^
ST-2G	89 ± 7.6 ^c^	1.30 ± 0.003 ^h^
ST-2H.+	71 ± 7.9 ^cd^	1.40 ± 0.001 ^e^
ST-2I.+	57 ± 3.1 ^d^	1.44 ± 0.001 ^cd^
ST-2K.+	82 ± 14.7 ^c^	1.51 ± 0.027 ^a^
ST-2L.+	40 ± 3.4 ^ef^	1.39 ± 0.004 ^e^
ST-2M	75 ± 3.3 ^c^	1.43 ± 0.004 ^cd^
DSMZ20259	11 ± 2.4 ^gh^	1.31 ± 0.008 ^gh^

^1^ A ‘+’ after the strain code indicates microscopic evidence of cell-bound EPS. ^2^ Mean values (± half deviation range, *n* = 2) with different superscripts per column differ significantly (*p* < 0.05).
